# Positive Health and the happy professional: a qualitative case study

**DOI:** 10.1186/s12875-021-01509-6

**Published:** 2021-07-24

**Authors:** Caro H. C. Lemmen, Gili Yaron, Rachel Gifford, Marieke D. Spreeuwenberg

**Affiliations:** Department of Health Services Research, Care and Public Health Research Institute (CAPHRI), Faculty of Health Medicine and Life Sciences, Maastricht University, Duboisdomein 30, PO-Box 6220MD, Maastricht, 6229 GT The Netherlands

**Keywords:** Positive Health, Physician well-being, Primary care, Job satisfaction, Narrative identity theory

## Abstract

**Background:**

Primary care professionals (PCPs) face mounting pressures associated with their work, which has resulted in high burn-out numbers. Increasing PCPs’ job satisfaction is proposed as a solution in this regard. Positive Health (PH) is an upcoming, comprehensive health concept. Among others, this concept promises to promote PCPs’ job satisfaction. However, there is limited research into PH’s effects on this topic. This study, therefore, aims to provide insight into how adopting PH in a general practice affects PCPs’ job satisfaction.

**Methods:**

An ethnographic case study was conducted in a Dutch general practice that is currently implementing PH. Data collected included 11 semi-structured interviews and archival sources. All data were analyzed thematically.

**Results:**

Thematic analysis identified three themes regarding PCPs’ adoption of PH and job satisfaction, namely [1] adopting and adapting Positive Health, [2] giving substance to Positive Health in practice, and [3] changing financial and organizational structures. Firstly, the adoption of PH was the result of a match between the practice and the malleable and multi-interpretable concept. Secondly, PH supported PCPs to express, legitimize, and promote their distinctive approach to care work and its value. This strengthened them to further their holistic approach to health and stimulate autonomy in practice, with respect to both patients and professionals. Thirdly, the concept enabled PCPs to change their financial and organizational structures, notably freeing time to spend on patients and on their own well-being. This allowed them to enact their values. The changes made by the practice increased the job satisfaction of the PCPs.

**Conclusions:**

PH contributed to the job satisfaction of the PCPs of the general practice by functioning as an adaptable frame for change. This frame helped them to legitimize and give substance to their vision, thereby increasing job satisfaction. PH’s malleability allows for the frame’s customization and the creation of the match. Simultaneously, malleability introduces ambiguity on what the concept entails. In that regard, PH is not a readily implementable intervention. We recommend that other organizations seeking to adopt PH consider whether they are willing and able to make the match and explore how PH can help substantiate their vision.

**Supplementary Information:**

The online version contains supplementary material available at 10.1186/s12875-021-01509-6.

## Background

In response to the negative impact of high burn-out numbers, healthcare organizations and policymakers are increasingly showing interest in professional well-being, particularly in primary care [1–6]. Accordingly, research into professional well-being has surged. Whereas earlier scholarship focused on job dissatisfaction and burn-out, studies now mostly focus on such concepts as job satisfaction, joy in practice, and job enjoyment [4, 7, 8]. This article will focus on job satisfaction, as this term is used most in the research literature.

**Fig. 1:**
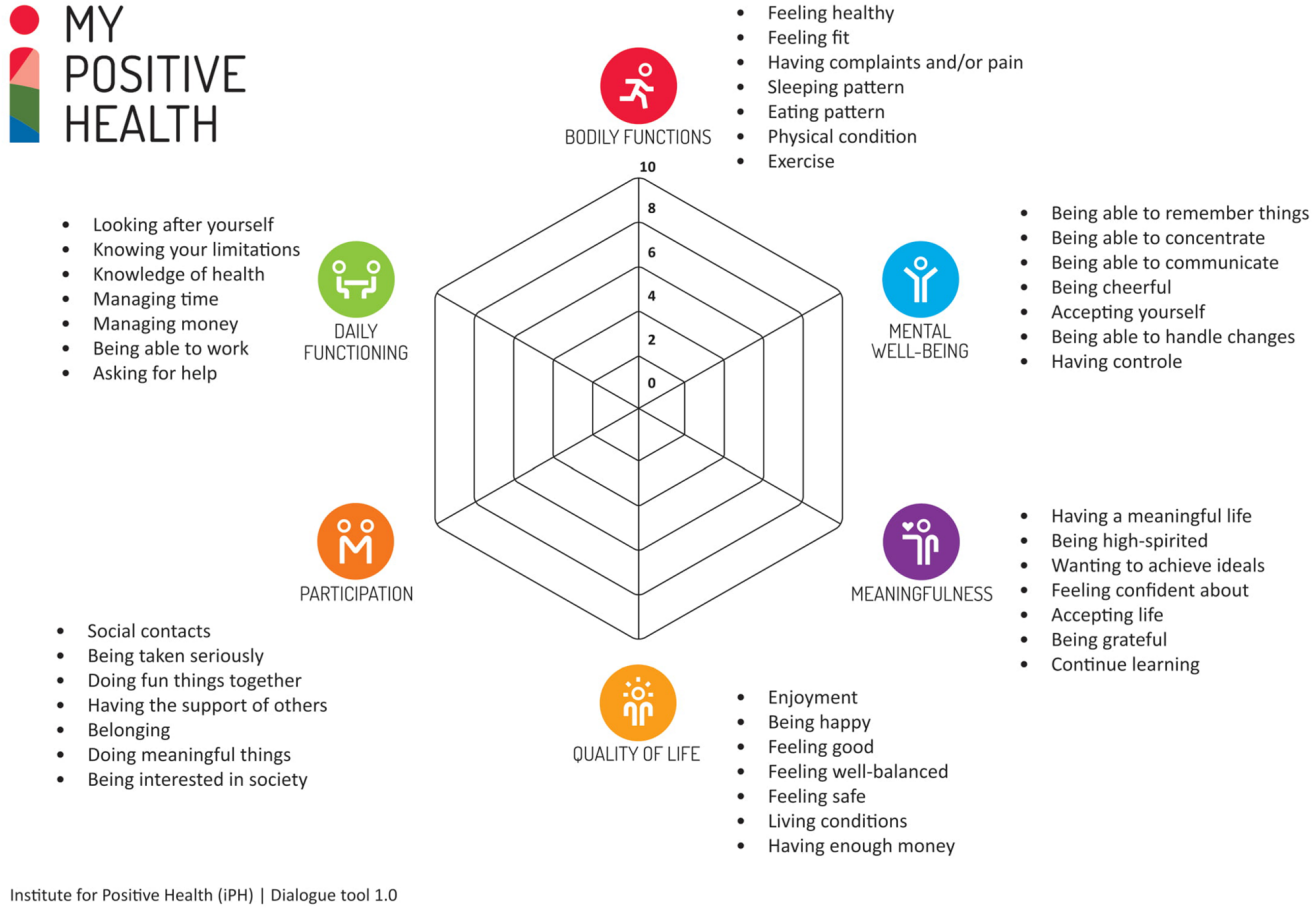
The six dimensions of Positive Health are visualized in ‘the spiderweb’ From: The institute for Positive Health [9]

Positive Health (PH) is a prominent, upcoming health concept comprising six dimensions relevant to experienced health (Fig. 1) [10]. PH operationalizes the new description of health proposed by Huber and colleagues in 2011 [11, 12]. According to this description, health is “the ability to adapt and self-manage in the face of social, physical, and emotional challenges” ([12], p1). In the last decade, Huber and others have worked towards developing tools for the concept’s implementation, most notably through the institute for Positive Health established in 2015 [9, 11, 13]. The most prominent application is a dialogue tool (‘the spiderweb’) intended to guide interactions between health professionals and patients.

Recently, several Dutch policymakers, professionals, and organizations have adopted PH as a promising way to tackle several issues in healthcare. The concept is praised for its promise to support patient autonomy, incorporate aspects of life beyond bodily functions in patient care, and promote cooperation between different fields, such as healthcare and welfare. Additionally, proponents emphasize PH’s potential to increase professionals’ job satisfaction [14–16]. This is linked to the concept’s purported effect on their autonomy and work-related meaningfulness. Opponents have criticized the concept for its vague boundaries, limited added value to the profession, and potential to increase health inequalities [17–20].

Meanwhile, the dearth of studies exploring the implementation of PH in practice makes it difficult to grasp the concept’s impact on job satisfaction. At present, there are a number of anecdotal reports by Dutch primary care professionals (PCPs) on PH’s positive effect in this regard [13, 14, 21]. In the context of a general practice, one recent study showed that working with PH, in combination with longer consultations and less patients per general practitioner (GP), led to more job satisfaction [22]. The present article aims to help fill this hiatus. Drawing on an ethnographic case study into the experiences of PCPs who work with PH in a general practice, it provides further insight into the concept’s effect on PCPs’ job satisfaction. Before we detail our methodology and discuss our results, it is prudent to first offer an overview of primary care in the Netherlands and of the literature on job satisfaction.

### Primary care in the Netherlands

General practices play a central role in Dutch primary care and healthcare in general because of their gatekeeping function [23, 24]. These practices have seen major reforms in the last 15 years. In 2006 a system of managed competition was introduced for curative care, which means that health insurers contract general practices for the provision of care. Moreover, general practices have received more responsibilities, including provision of certain specialist care tasks, mental health support, and coordination of community care [22, 23, 25]. As most Dutch general practices are owned by a partnership of GPs which employs other PCPs, these changes emphasized the role of GPs as both physicians and entrepreneurs [23–25].

These changes have had a major impact on PCPs’ work. Firstly, business thinking and practices, such as efficiency indicators and standardization through protocols, have entered primary care [26–28]. Professionals have been resisting these changes, as they challenge professional autonomy and occupational control of work that defines professionalism [26–30]. Additionally, multidisciplinary collaboration within and beyond the general practice has become increasingly important to cope with the additional care tasks [23, 24, 31]. This has also led to a shift in responsibilities from the GP to other PCPs. For instance, general practice-based nurse specialists have taken over tasks such as care for chronic conditions and mental health support [24, 25, 32]. Therefore, PH’s promise to integrate different fields (and thus ease collaboration between PCPs) and to increase professional autonomy corresponds with challenges in the changing primary care environment [15].

### Job satisfaction

Research into job satisfaction is heavily influenced by Herzberg’s two factor theory. Herzberg and colleagues hypothesized that factors intrinsic to the content of the job, such as received recognition, autonomy, and personal growth have a positive influence on job satisfaction. By contrast, a deficiency in factors extrinsic to the actual work, such as interpersonal relationships, working conditions, salary, organization policy, and administration are assumed to decrease job satisfaction [33, 34]. Several studies into job satisfaction of PCPs confirmed these hypotheses [4, 35–38].

The divide between intrinsic and extrinsic factors can also be seen in the literature on physician well-being interventions. Examples of extrinsic interventions are workflow improvements, individual resilience courses, or communication training [39–41]. Interventions focusing on intrinsic factors aim to create joy through meaning-making, defining joy as a “feeling of success and fulfilment that results from meaningful work” ([8] p608). To engage professionals, authors propose helping them reflect on their professional identity via questions such as: ‘why did you choose this profession?’ or: ‘what calls you to practice?’ [35, 42–44]. Consequently, part of PH’s appeal is that it explicitly includes meaning-making as one of its six dimensions for both patients and professionals. Several healthcare providers reported that PH helped them find meaning in their work [15, 42, 45], however the impact of PH on meaning-making by professionals has not yet been empirically studied.

## Methods

### Research aim, design, and setting

This article is based on an ethnographic case study into how the adoption of Positive Health affected primary care professionals’ job satisfaction. The present study focused on one general practice that was implementing PH. All twelve staff members were asked to participate in the study to explore how PH affects the various professions, interprofessional relationships, and practice as a whole. In order to gain an in-depth understanding of PCPs’ experiences, the first author collected archival material and conducted qualitative online interviews with PCPs.﻿ 

### Data collection

Archival materials collected included documents associated with the implementation of PH in the general practice, the general practice itself, and the history and development of PH. These documents included, among others: minutes from team meetings, internal management documents, communication between the practice and the health insurer, the website of the general practice, and newsletters. Documents selected for inclusion were written either in Dutch or English.

Interviews took place in April and May 2020 and the researchers employed a semi-structured approach. This allowed for flexibility in pursuing an interviewee’s experiences, while ensuring that all topics were addressed [43]. To this end, the researchers constructed an interview guide to cover the implementation of PH, job satisfaction, and professionalism (Additional file 1) [43]. Before the start of the interviews, the first and second author met with the practice manager and GPs to ensure transparency and align mutual expectations. Interviews were carried out via a licensed version of Zoom Video Communications, Inc. and once by phone, considering safety measures taken in response to the COVID-19 pandemic. All interviews were audio recorded. To increase the reliability and validity of the study, the researchers made use of member checks by offering the participants a summary of their interview [44]. Written informed consent for publication was obtained from all participants.

The interviews were carried out by the first author. At the time of the study she held a Bachelor of Science degree and was a master’s student at Maastricht University, as well as a medical student at Utrecht University. She had limited experience with respect to interviewing. To ensure the quality of the project, the entire process was supervised and guided by the second author, a senior researcher at the department of Health Services Research of Maastricht University. She is an experienced qualitative researcher with expertise in healthcare humanities and science & technology studies.

In total, approximately 270 pages of material on this general practice, and 250 pages on the history and development of Positive Health were analyzed. Eleven out of the total of twelve primary care professionals were able to participate in the interviews (Table 1). One employee declined because of personal circumstances and upon request the personal data of one respondent will not be disclosed. The interviews lasted on average 58 min. Each participant was interviewed once. Ten participants were present in the workplace during the interviews, one participated from home. The interviewer was based at her home. No non-participants were present during the interviews. The interviewer took notes during and after the interview. Interviews ended when all participants were included.

**Table 1:** Overview of respondents

**Respondent**	**Profession**	**Gender**	**Age**
**1**	Practice manager	Female	49
**2**	Practice nurse	Female	46
**3**	Practice nurse	Female	59
**4**	Practice-based nurse specialist	Male	57
**5**	General practitioner and practice owner	Male	40
**6**	General practitioner	Female	35
**7**	X	X	X
**8**	Practice nurse	Female	26
**9**	Practice nurse	Female	57
**10**	Practice-based nurse specialist	Male	58
**11**	General practitioner and practice owner	Male	49

### Data analysis

Analysis was conducted iteratively by the first, second and third author. In analyzing interviews and documents, the researchers followed an inductive approach using the thematic analysis guide of Braun and Clarke [46]. NVivo 12, a qualitative analysis software program, was used to keep track of the analysis. Thematic analysis consisted of an iteration of three phases of coding, namely open, axial, and selective coding [46]. During the coding process, the first and second author regularly discussed the proposed codes and themes to reach consensus. The third author aided the first and second author in establishing themes and subthemes. The COREQ checklist was used when writing this report (Additional file 2) [47]. The results were presented to the participants, who recognized themselves in the analysis.

## Results: Positive Health as an adaptable frame for change

Thematic analysis identified three themes regarding the adoption of Positive Health and primary care professionals’ job satisfaction, namely [1] adopting and adapting Positive Health, [2] giving substance to Positive Health in practice and [3] changing financial and organizational structures. Theme 1 describes how the adoption of PH in this practice was the result of ideology, strategy, and favorable circumstances. It also discusses the multi-interpretability and malleability of PH. Adopting PH enabled PCP’s to better express, legitimize, and promote their values of holism and autonomy. These values were not only prominent in patient care, but also in the attitude of professionals towards each other, which is addressed in theme 2. This supported them to align everyday conduct with how they envisioned their profession, which increased their job satisfaction. Next, theme 3 depicts how the practice changed its financial and organizational structures upon adopting PH. Consequently, PCPs could invest more time in their patients and in their own well-being. They, were, thus, able to further their vision in practice. These changes contributed to their job satisfaction. In this way, PH functioned as an adaptable frame for change in the general practice. The following pages will unpack this conclusion. An additional data table with quotes is provided in Additional file 3.

### Adopting and adapting Positive Health

Most respondents considered the adoption of Positive Health to be a minor change from former ways of working, since PH fit the vision of the practice well. However, several respondents explained that precisely this match made the adoption of the concept possible. In addition, PH provided the practice with a new frame to express and legitimize its comprehensive approach to healthcare. At the same time, the respondents offered different interpretations on what the concept entailed and the practice adapted the concept to its needs in several ways. This section will further unpack how the practice adapted itself to adopt PH, and adapted the concept while adopting it.

#### It’s a match!

Document and interview analysis showed that the introduction of PH in this practice was the result of ideology, strategic considerations, and fortunate circumstances. The current practice owners dissociated from another general practitioner in 2015 due to a personal conflict, different ideas about the role of the GP, and other styles of practicing. The newly emerged practice aimed to interact with patients on a more personal level, to approach them holistically, stimulate their self-reliance, and to be part of the community. One GP described the role the practice aspires to fulfill in the community:

*I would also like it if [the villagers] just say: ‘it’s a place where I like to go (…) that general practice, you can do everything there (…) you should go there to talk about your stairlift or about your cleaning service, or depressed spouse’, and that we don’t immediately say: (…) ‘that’s not the job of the GP’, but that we say: ‘come let’s look at it together’ and of course immediately involving (…) [our] network.* (R11, GP)

In pursuing this vision, the practice made several changes. For instance, they created a volunteer-run vegetable garden and raised the minimal consultation time from 10 to 15 min to create more time for the patients. With this, the practice owners aimed to create more joy^1^ in their work, as this nurse specialist explained:

*They made the choice a couple of years ago themselves, also because of their job enjoyment, to do things differently. So they conscientiously chose to spend more GP hours than they formally could according to the guidelines, in order to have more job enjoyment and more time for the patient.* (R4, nurse specialist)

The practice’s alternative way of working was met with interest from a health insurer that was looking to experiment with Positive Health as a new health concept. Conversations between the practice and health insurer started in 2017, followed by the practice adopting the concept of PH. This helped the practice to express and legitimize its new approach to care work:

*[Y]ou can say ‘we’re working in conformity with Positive Health.’ ‘Oh right, I have quite a good picture of what that entails at yours then.’ Plus, it can be an argument to partners and financiers to get done what you want.* (R10, nurse specialist)

In 2019 a pilot project under the flag of PH started between the practice, the insurer, and the municipality. One of the goals of the pilot project was to improve job satisfaction, which prompted this study. Theme 2 and 3 will provide further detail on how the practice implemented the concept.

Interestingly, some participants pointed out that this match between a general practice and PH is not self-evident. One of the GPs explained why the concept is not a fit for everyone:

*There are also a lot of people who would not like this at all. They say, that’s just drivel, that’s not at all family medicine. But well, I have noticed that I do like it. I don’t feel like I’m being less of a doctor. Instead I get more to the core.* (R11, GP)

Explaining this, he said, that some GPs would consider this approach not medical enough; talking about the person rather than the complaints.

#### Positive Health as a malleable concept

Despite the small size of this practice, the PCPs offered different interpretations of PH during the interviews. Some respondents struggled to describe what the concept meant:

*I still find it hard to give it something tangible, like what is it really? In fact it is a little bit of everything in your work.* (R2, practice nurse)

Two new employees reported that they had not received information on what PH entailed or how it should be implemented when they started working in the practice. However, professional values that hang together with PH (e.g. collegiality, the importance of self-care), were discussed more elaborately. Interestingly, the practice’s policy documents lack a clear-cut description of how the practice owners interpreted PH or envisioned the concept’s implementation. This could explain the conceptual confusion amongst the employees.

Analysis of interviews and archival material reveals four different descriptions of the essence of PH. Most commonly, working according to PH was described as taking a holistic or person-centered approach. The second most mentioned interpretation of PH was focusing on patient autonomy and resilience. The third interpretation of PH saw it as focusing on health and possibilities instead of illness and limitations. A fourth interpretation of PH found in the data described it as a way to achieve professional well-being:

*I think it’s a way of working within your team (…) focused on living as healthily as possible (…) and also extending that to your patient care.* (R7, X).

How the PCPs applied these interpretations in their work will be discussed in theme 2 and 3.

Notably, employees mentioned lifestyle-related issues in relation to all four different interpretations of PH, illustrating the importance of lifestyle in the general practice. This focus on lifestyle is seen in publications by and about the practice; however, other organizations adopting PH, did not put such a marked emphasis on lifestyle. This variance suggests PH is a malleable concept, offering room for multiple interpretations and allowing for context-specific customization.

### Giving substance to Positive Health in practice

In line with difficulties in interpreting PH, some participants struggled to describe how they gave substance to the concept in practice. Several participants considered the practice’s public story to be changed the most. This included how the practice promoted its vision and how PCPs now stimulated each other to adhere to a healthy lifestyle. But upon taking a closer look, more implicit changes appeared to have occurred as well. The concept’s focus on holism and autonomy, towards both patients and professionals, strengthened the PCPs’ commitment to these values. In fact, PH served as legitimization for the distinct approach of the practice: PCPs felt supported to align their everyday conduct with their vision, which, as many said, contributed to their job satisfaction (Additional file 3). How the participants gave substance to their profession and to PH in practice will be described in the following paragraphs.

#### Consolidating the practice’s vision

Notably, when asked what changed most when the practice adopted PH, several respondents suggested it was the way the practice presented its vision and values. Indeed, the practice abundantly communicated its unique approach to family medicine in newsletters and on the website. PH and the 6-dimensional PH model took a prominent place in all these communications, thereby becoming absorbed in the practice’s public story. As the following quote illustrates, PH offered the practice a frame to express and promote its vision:

*Something that I’ve been personally working with, now (…) becomes embedded. ‘Ah right,* *that’s* *what it’s called!’ And that (…) provides a frame to relay it to others.* (R1, practice manager).

A majority of participants explained that the changed public image included setting the right example with respect to their own well-being. For instance, the participants had healthy lunches together or went on walks in the village during lunchtime. Most enjoyed this way of working.

Symbolizing the practice’s vision, the vegetable garden appeared in nearly all publications by and about the practice. Apparently in this way the garden functioned as a visual reminder of the importance of lifestyle and community-outreach in their vision:

*We have been working for a long time on propagating and demonstrating a healthy lifestyle and have done this up and until now mostly in 1 on 1 patient consultations, in our offices. In the vegetable garden project we want to step outside and play a part ourselves by making the garden/ grounds available and making the first move.* (Internal document; Project Proposal Vegetable Garden)

In the interviews, too, several respondents mentioned the garden in relation to the practice’s vision and adoption of PH.

By adopting PH, the practice saw itself as part of a movement in the healthcare field. Document analysis showed that the practice hoped to become an inspiration for other organizations and contribute to a positive change in the healthcare field. This aspiration also came to the fore during a few interviews:

*I think that it [Positive Health] will grow. I think that considering society wants to go there, we’re on the right track. And by then we will become an example for multiple practices that will come and ask us: ‘Gosh, how are you doing that? How do you tackle that?’* (R9, practice nurse)

Through these various communications the practice positions itself as being on the cutting edge of innovation.

#### Towards a holistic approach

Even though several participants initially referred to the practice’s changed public story as the main effect of PH, during the analysis it became clear that more implicit changes had occurred as well. The adoption of PH increased the PCPs’ commitment to approaching patients and themselves holistically, to stimulating the autonomy of both, as well as to giving substance to this in practice:

*Embracing the concept of Positive Health creates space in the practice for (…) egalitarian relationships, the autonomy you get as a professional, possibilities to tune in, giving substance to consultations and office hours how you think is nicest. I think that within this practice they would have done this without a concept like Positive Health. But, it is nice that Positive Health as an idea exists, so that you can use it as a frame for what the practice was doing already.* (R10, nurse specialist)

As this quote shows, PH created space for the PCPs to better align their daily practice with how they envisioned their work. It also supported them in legitimizing this approach to others, some PCPs explained. Especially GPs and nurse specialists pointed out that this increased their job satisfaction.

All participants expressed the importance of a person-centered and holistic instead of a provider-centered and problem-oriented approach in patient care, which was also stressed in the practice’s policy documents and external communication. During patient consultations, GPs and nurse specialists strove towards having an open conversation and addressing issues beyond bodily complaints if needed:

*I don’t ask a patient: ‘So, I see you are coming with knee complaints. What’s going on with your knee?’ Instead, I ask a patient: ‘How are you?’ Or: ‘Tell me?’ Or: 'How are you doing?’ So you start much more broad.* (R6, GP)

They aimed to be open to all kinds of problems and to have eyes for the broader context of the patient. For example, one GP points out that she called the Dutch employee insurance agency, because she was worried that the lack of financial stability affected a patient’s health. The practice’s holistic approach expanded the borders of the consultation room and involved the whole team. The practice nurses checked in with every patient that returned home from a hospital admission, and the GPs regularly visited the elderly in the neighborhood to see how they were doing.

To strengthen their holistic approach, the PCPs participated in a PH training. The evaluation report of this training showed that the GPs were unsure how and when to embed the 6-dimensional PH model as a dialogue tool in their consultations, which they confirmed during the interviews. Instead, the GPs viewed PH primarily as a foundational attitude to facilitate an open exchange with patients:

*But the idea [behind PH] is that you get to know the person behind the patient (…) What makes them tick, if you go searching for that, well that can bring a lot, because then you just have a different conversation (…) less medical and more human or something (R11, GP).*

In fact, they rarely use the PH dialogue tool in consultations. Rather, they choose to reserve it for situations in which they feel the patient could benefit from more insight regarding the issue at hand.

As some respondents pointed out, this approach contrasts sharply with the conventional provider-centered conversation, in which PCPs ask steering questions. However, this approach was not new for all participants. The nurse specialist for mental health, for instance, argued that this type of conversation was already commonplace in mental healthcare. In addition, some assistants concluded that their patient interactions had always been more personal and less medical when compared to that of the GPs, and, in fact, had not been changed by PH:

*It’s more the physicians who use it [Positive Health] the most. And how it really shapes us assistants, I cannot specifically say. It’s more like we already did before, giving attention to the patient. (R2, assistant)*

#### Towards more autonomy for both patients and professionals

In conversations with patients, the GPs and nurse specialists tried to stimulate patient autonomy by exploring patients’ wishes and values, as well as by supporting them on how to realize these. To illustrate this, the practice manager discussed the case of an elderly woman just diagnosed with metastasized cancer:

*[The GP] was immediately told by the hospital these [are] the treatment options. And in the conversation with that woman, whom he has known already for a while and he knows her partner, she decided (…) I want quality of life that’s more important to me now than that I might live a half year extra.* (R1, practice manager)

As this quote illustrates, the GP in question focused on the patient’s preference rather than the available treatment options. Some other respondents also described how they addressed lifestyle-related issues but left it to the patient to make a follow-up appointment or decide on the treatment plan. This indicates a move towards more egalitarianism in the relationship between patient and professional, including a more directive role for patients in shaping the care process.

As shown previously, supporting patient autonomy was a significant part of the respondents’ understanding of what PH entails. For example, one GP sometimes used the 6-dimensional model as an instrument and asked patients to fill it in at home:

*If you have the idea that people could use more insight, more control over their own health or over their own life in a broader sense, then I think it’s a convenient instrument to bring forward.* (R5, GP)

This approach contrasted from the other GPs in the practice, who underscored the importance of stimulating self-reliance, but did not use the 6-dimensional model for this.

Most respondents expressed how stimulating patient autonomy and taking a holistic approach increased their job satisfaction:

*What contributes a lot to my job enjoyment is that (…) it’s a unique patient who sits in front of me every time. (…) I always say to people like ‘you are unique, so this is the protocol, but we have to check with you together what constitutes tailor-made care here. ‘What can you do? What do you want? What’s not possible? What is possible?’ To figure that out together. I like that very much.* (R4, nurse specialist)

As this quote illustrates, working like this allowed the PCPs to focus more on the person than on the conventional biomedical protocol. This increased the space they experienced to provide what they consider good care and figure this out together with the patient. They themselves indicated this was challenging, but increased their professional autonomy. In addition, some PCPs also explained that this way of working allowed them to connect better with patients, which positively affected their job satisfaction. However, it was unclear to most PCPs what the exact influence of PH was on their patient relationships.

### Changing financial and organizational structures

Upon adopting PH, the practice also altered financial and organizational structures through the aforementioned pilot project with the health insurer and municipality, as was described in the project plan, the minutes of project meetings, and communication with the health insurer. The changes resulted in extra time for PCPs to spend on patients and on themselves. Several PCPs also expressed that the additional time increased their autonomy. Both the additional time and increased autonomy contributed to the job satisfaction of the participants under study (Additional file 3).

#### Money is time

An important part of the pilot project were new financial agreements made between the practice and health insurer: financing changed from activity-based contracts to capitation financing, and the practice received a higher staffing budget. As a result, the practice owners could lay aside income-related concerns:

*Well, [the contract with the insurance company] gives us peace and comfort to fully focus on what we think is good GP care in all its aspects.* (R5, GP).

Some PCPs reported that as a result of these new financial arrangements they felt less pressured in their daily work to perform activities that generate income.

The new way of financing supported the practice in their decision to hire more staff so as to prolong consultation times and lower the pace of their daily work. All assistants reported that reduced work pressure and more time for patient interactions increased their job satisfaction:

*The freedom you have to really be there for the patients. Because I think that’s wat makes it most fun. That you don’t have to rush a patient.* (R2, practice nurse)

Moreover, the additional time and lower work pace supported the practice in its endeavor to promote PH by setting a healthy example and investing in professional well-being, as described in theme 2.

The GPs and nurse specialists indicated that it was important for them to have sufficient time in order to carry out consultations in line with how they envisioned them. This enabled them to help patients how they thought was best. This includes taking the time to listen to the patient, discussing all domains of life, thinking along with a patient to find fitting solutions, and giving room for emotions. All of them explained that the feeling that they had helped patients was an important contributor to their job satisfaction:

*When they [the patients] enter with their head hung down, I want them to leave with their head held high. If possible I would like to add something to that. And I think that works best if you understand someone better. And then you will have to reserve a bit more time to figure that out*. (R11, GP)

Several PCPs explained that they not only enjoyed these interactions more, but also felt more autonomous. Both had a positive effect on their job satisfaction.

#### Easing collaboration

Secondly, as part of the pilot project the practice joined a regional project group working according to PH that consisted of the health insurer, municipality, welfare organizations, local associations, and volunteers. The goal was to create a network of various organizations around the general practice that could aid them in carrying out their community-focused and holistic approach. A GP summed up who is needed for this:

*In principle anyone that you can use to help the patient to get going. It can be someone from the social housing corporation, it can be a care path counselor, that can be the gardener.* (R11, GP)

With the project partners the practice carried out several initiatives to increase the health and well-being of the villagers. For instance, together they set out walking routes in the region with the practice as a starting point. With this, the practice and project group aimed to stimulate villagers to exercise. The practice organized lifestyle workshops for the entire village to help them adopt a healthy lifestyle. In addition, together with the municipality and social housing corporation the practice planned to build volunteer-supported living facilities for the elderly, inspired by PH.

The practice also sought collaboration within the healthcare field, for example with the home care organization or physiotherapists. Another example of this, was the start of multidisciplinary consultations with welfare and specialized mental healthcare professionals. However, only few participants explicitly stated that this eased collaboration contributed to their job satisfaction. One of them was this practice nurse:

*It’s nice that you’re also supported by other organizations so to say. That you’re not in it on your own. (…) I think eventually if you see that you’re a bit successful that gives joy in work. That you know what you’re doing it for.* (R8, practice nurse)

## Discussion

This study set out to explore how adopting Positive Health in a general practice affected the job satisfaction of primary care professionals. Firstly, the results show that the adoption of PH was the result of a carefully crafted match between the practice and the concept. Next, PH was shown to be a multi-interpretable and malleable concept. Secondly, PH supported PCPs to express, legitimize, and promote their distinctive approach to care work and its value. This created space to align everyday conduct with their vision, notably on the importance of holism and autonomy, which supported their job satisfaction. Thirdly, the concept enabled PCPs to change their financial and organizational structures allowing them to enact their vision, and improving their working conditions. These changes also increased the job satisfaction of the PCPs. In conclusion, PH acted as an adaptable frame for change, and in doing so contributed to the job satisfaction of the PCPs.

The changes positively affecting job satisfaction in the general practice resonate with the literature on job satisfaction and physician well-being interventions, which underscores that both intrinsic (e.g. meaning-making, values) and extrinsic factors (e.g. more time, context) play a significant role in this regard [4, 8, 35, 40]. Namely, sources of job satisfaction in this study were a decrease in financial and time pressures, allowing PCPs to invest time in their patients and in their own well-being. Furthermore, PCPs felt more autonomous in their profession and were able to act upon their own professional values of holism and autonomy. Additionally, previous research reported that taking a person-centered approach increased the job satisfaction of healthcare workers [36]. As several changes in the practice were initiated before the adoption of PH (e.g. prolonging consultation times, taking a person-centered approach) this raises the question how the concept contributed to the job satisfaction of the participants. Especially, as some of the respondents struggled to describe what the concept meant.

Indeed, tracing the contribution of PH to various changes within the practice, and understanding how each affected job satisfaction has proven a Sisyphean task during our analysis of the data. The concept’s multi-interpretability and malleability complicated this issue further, as they introduced ambiguity regarding PH’s content and application. For example, the PCPs under study rarely used the PH ‘spiderweb’ as a dialogue tool during consultations, even though the institute for Positive Health promotes such usage. Also the practice chose to give lifestyle a prominent place in its adaptation of PH, which contrasts how other organizations give substance to the new health concept. This ambiguity of PH has been noted previously by other organizations implementing the health concept [15, 19, 47]. Ambiguity can be a potential source of confusion and lead to a lack of uniformity in implementation.

However, our analysis indicates that the malleability of PH is a strength as well. Successful application of PH, in the case of the practice under study, hinged on a carefully crafted match between the concept and the organization. In fact, customization of PH permitted by the concept’s malleability, played an important part in the making of the match. The individual PCPs, as well, could develop their own way of adopting the concept. As described in theme 2, PH contributed to consolidating the practice’s vision, while allowing for individual variations in practice. Thus, the PH frame helped align PCPs with the organization’s vision and values without heavily infringing on the professionals’ autonomy. In this way, it contributed to PCPs’ job satisfaction [35, 40, 48].

Over the past years, PH’s popularity has been soaring in the Dutch health landscape. The comprehensive health concept appears in publications by the national government, the association of GPs, and many municipalities, for instance [13]. Thus, PH’s recognizability and legitimacy in the Netherlands allowed the practice to use the concept as a frame for change to express, legitimize, and promote its vision. The adoption of this frame enabled the emergence of an experimental project with the health insurer and municipality. As such, PH helped secure the material (most importantly financial) conditions needed for the practice’s preferred way of working, namely for PCPs to invest more time in their patients and in their own well-being. The legitimacy of the frame also supported PCPs to give substance to their vision in practice. As discussed, these changes positively affected the PCPs’ job satisfaction. Arguably, PH fulfills a similar framing function in comparable experiments in the healthcare field [15, 21, 22, 48].

Moreover, we argue that the framing function of PH contributed to the meaning-making of individual PCPs. A frame strengthens the coherency and value of one’s identity or ideas, for instance, on professionalism, which according to narrative identity theory makes individuals feel validated and authentic [49–52]. Put more concretely, a frame helps conveying what someone stands for, what makes them proud, and how they imagine the future. As theme 2 demonstrates, PH endorsed the PCPs in this regard. Due to the malleability of PH, each PCP could adopt and adapt it in such a way that the frame fit their professional identity. Thus, we argue that PH furthered the PCPs’ job satisfaction by supporting them to find meaning and a sense of purpose in their profession [4, 35]. Anecdotal accounts by healthcare professionals suggest that PH has contributed to their meaning-making [15, 16, 42, 45]. So far, however, the influence of PH on meaning-making in practice has not been addressed in the research literature, nor sufficiently by the current study. Therefore, future research should explore the role of PH on meaning-making and how this relates to job satisfaction.

Our results suggest that each organization that seeks to introduce PH as an intervention to improve job satisfaction will need to invest significant effort in making the match. Therefore, we recommend that other organizations first explore whether they are willing and able to do so. Nevertheless, adopting PH can be taken as an opportunity for professionals and the organization to start discussing relevant topics relating to the concept and to job satisfaction. These include the meaning of professionality, values such as holism or autonomy, and the organization’s place in the community. All the while, organizations should facilitate concrete changes that resonate with the vision developed (e.g. financial rearrangements, lunchtime walks). This case report shows that investing in PH can help legitimizing and giving substance to these changes, thereby potentially improving job satisfaction. Recently, the institute for Positive Health published guidelines on how to implement PH in a general practice [53]. In line with our recommendations, the authors stress the importance of reflecting on how PH can contribute to a practice’s vision and values, before making changes in the organization.

This study has several strengths and limitations. Since it is a case study, only a limited number of professionals were interviewed in a specific setting. Indeed, the discussed match is not possible in each and every setting. This minimizes the generalizability of the results. Additionally, the study population has an outspoken positive attitude towards PH, which might have introduced confirmation bias. Moreover, the participants are stakeholders in this research, as this study was used to evaluate the effects of PH on PCP well-being in the general practice. Therefore, the results may have an impact on the continuation of the pilot project with the municipality and health insurer. As this could have led to a conflict of interest, all stakeholders, including the researchers, met to discuss expectations and stakes at the start of the study. Consequently, agreements on transparent communication were made. The strong cooperation with the practice also meant a multitude of in-depth data and insights could be gathered, which increased the validity of the study [44]. Finally, the study forms a unique addition to the scarce academic research on PH implementations and professionals’ meaning-making in healthcare.

## Conclusions

Positive Health contributed to the job satisfaction of the primary care professionals of the general practice by functioning as an adaptable frame for change. Firstly, the frame aided them in expressing, legitimizing, and promoting their vision on good healthcare and professionalism, and its value. This supported the PCPs to align everyday conduct with their values. Secondly, PH served as a frame to secure material conditions that were needed to enact this vision, notably freeing time to spend on patients and invest in PCP’s own well-being. However, it is important to take into account that the practice’s vision and circumstances fit well with the concept of PH, and vice versa. It is not self-evident that such a match is possible in any given context. Additionally, PH’s malleability introduces ambiguity in what the concept entails, but also allows for context-specific customization. In that regard, PH is not a readily implementable intervention. We recommend that other organizations seeking to adopt PH to increase job satisfaction carefully consider whether they are willing and able to make the match and explore how PH can contribute to substantiating the vision and values of the professionals involved. Future studies should explore the role of PH on meaning-making and how this relates to job satisfaction.

## Supplementary Information

**Additional file 1.** Interview Guide. Guide used during the semi-structured interviews.

**Additional file 2.** COREQ (COnsolidated criteria for REporting Qualitative research) Checklist. COREQ Checklist describing items that should be reported in qualitative research manuscripts and on which page to find these in the manuscript.

**Additional file 3.** Additional Quotes Table. Data table providing a selection of additional quotes per theme.

## Data Availability

The datasets generated and analyzed during the current study are not publicly available due to privacy concerns but are available from the corresponding author on reasonable request.

## Abbreviations

PH: Positive Health
PCP: Primary care professional
GP: General practitioner

## Footnotes

1 The participants themselves spoke of job enjoyment or joy in work in their interviews as opposed to job satisfaction. These terms will, therefore, be used when quoting or paraphrasing participants.
