# Supplementary material for: Positive Health and the happy professional: a qualitative case study
Source: BMC Fam Pract. 2021 Jul 24;22:159. doi: 10.1186/s12875-021-01509-6 (PMC8308069; doi:10.1186/s12875-021-01509-6)
Supplement: Supplementary file 1 — Additional file 1. Interview Guide. Guide used during the semi-structured interviews. [file 12875_2021_1509_MOESM1_ESM.docx]

**Interview guide**

| **Introduction** | | | | | | | |
| --- | --- | --- | --- | --- | --- | --- | --- |
| Thank you. Is it okay if I speak informally? I realize some topics might be sensitive.  I would like to start by telling something **about this study**. This study is part of my master’s thesis at Maastricht University and might be published in the future. Next tot this, the study is part of a bigger research project into the dissemination and implementation of PH in Limburg by my thesis supervisor, Dr. Gili Yaron. She might use these interviews for her research too.  **Aim of the interview**: Insight in the impact of working with PH on the job enjoyment of all practice employees. Next to that, I’m interested in specific factors that influence job enjoyment, such as patient interaction, professionalism, and teamwork.  **Practicalities**: Duration of the interview: max 1 hour. Then, recording and transcribing, taking into account confidentiality. Pseudonyms in publications. Next step: Summary & member check & potential follow-up questions: ok? Next, thematic analysis. Questions on informed consent? With respect to online videocalling: Awkward moments, silences, let each other finish as much as possible, facial expressions/ gestures. Questions? Start recording. | | | | | | | |
| **Background** | | | | | | | |
| For starters I would like to know more about your background. Can you tell me something about that? | | | | | | | |
| What did you do before you started working at [practice]? | | | | | | | How did you end up at [practice]? How long have you been working here? |
| **Positive Health** | | | | | | | |
| Moreover, I’d like to ask a couple of questions about Positive Health to gain more insight into your experiences with this concept. How did you first learn about Positive Health? | | | | | | | |
| What is your opinion on the concept Positive Health? | | | | | | | |
| *What does PH mean to you?* | | | | | | | |
| The practice has agreements with [organizations] since 1 January 2019. How did these agreements come about? | | | | | | | |
| *Can you tell me more about these agreements? What exactly do they entail?* | | | | *How was the idea received by other employees in the practice?* | | | *What happens if the agreememnts with [insurance company] aren’t prolonged?* |
| According to you, what has changed the most in the practice since you started working with Positive Health? | | | | | | | |
| All employees have received a PH training and some have started specializing in the concept. Can you tell me a bit about this? | | | | | | | |
| How do you incorporate Positive Health in your daily work? | | | | | | | |
| What do you consider advantages of working with Positive Health in the general practice? | | | | | | | |
| What barriers do you experience while working with Positive Health? | | | | | | | |
| *What do you need to work in accordance with Positive Health?* | | | | | | | |
| **Job enjoyment** | | | | | | | |
| Like I just explained, the goal of this research is to look into the impact of Positive Health on your job enjoyment or job satisfaction. I, therefore, wonder what do you think contributes most to your job enjoyment? | | | | | | | |
| What makes that you don’t feel like going to your work sometimes? What are things that make you enjoy your work less? | | | | | | | |
| How would you describe your satisfaction with your work at this moment? | | | | | | | |
| *How was this in the past?* | *What has been the impact of working with PH on this?* | | | | | | *Can you give examples of this?* |
| How do your colleagues experience working with Positive Health? | | | | | | | |
| The literature shows a connection between job enjoyment and leave of absence. How do you see this? | | | | | | | |
| *What has been the impact of working with PH on this?* | | | | | | | *Can you tell me something about absenteeism at [practice]?* |
| **Patient interaction** | | | | | | | |
| Research shows that good patient interaction is an important contributor to job enjoyment of healthcare professionals. How would you describe good patient interaction? | | | | | | | |
| *Do you have examples of this?* | | *What’s the influence of this on your job enjoyment?* | | | | | *What has been the impact PH on this?* |
| **Teamwork** | | | | | | | |
| Next to this, teamwork seems an important factor in the job enjoyment of people. How do you experience this? | | | | | | | |
| *Do you have examples of this?* | | | *Why is that?* | | | | *What has been the impact of working with PH on this?* |
| I frequently read in the documents from the practice that you consider a positive atmosphere in the team very important. How do you make that happen? | | | | | | | |
| *Why do you think that’s important?* | | | | | *What has been the impact of PH on this?* | | |
| **Professionalism** | | | | | | | |
| How do you shape your profession as [profession]?  *What do you consider to be important in your job as [profession]?*  *What makes someone a good [profession]?* | | | | | | | |
| *How free are you in shaping your own activities as [profession]?* | | | *Do you have examples of this?* | | | *What does it mean to you to carry out your profession well? What makes you get out of your bed in the morning?* | |
| Lastly, I’m curious what the effect is of working with PH on how you view your profession as [profession]. Can you tell me something about that? | | | | | | | |
| *Do you have examples of this?* | | | *What has changed?* | | | | *Why?* |
| **Closing** | | | | | | | |
| Finally, I would like to ask you a last question about Positive Health. What do you consider the core of Positive Health? How do you imagine the future of the practice with regards to Positive Health? | | | | | | | |
| Things you would like to add that haven’t been discussed? | | | | | | | |
| What did you think of the interview? | | | | | | | |
