# Supplementary material for: Positive Health and the happy professional: a qualitative case study
Source: BMC Fam Pract. 2021 Jul 24;22:159. doi: 10.1186/s12875-021-01509-6 (PMC8308069; doi:10.1186/s12875-021-01509-6)
Supplement: Supplementary file 3 — Additional file 3. Additional Quotes Table. Data table providing a selection of additional quotes per theme. [file 12875_2021_1509_MOESM3_ESM.docx]

**Additional Quotes Table**

Table 1 A selection of additional quotes per theme

| Theme 1: Adopting and Adapting Positive Health | | |
| --- | --- | --- |
| *On how Positive Health matches their vision/ ideas* |  | |
| [On working with PH] It’s exactly how I also look at disease myself. (…) And here you find that. Here they work with this. Then I’m like: ‘Wow great, good job!’ | R3, practice nurse | |
| We are not a Positive Health practice. But we embrace, let’s say, the body of thought of Positive Health within our practice, because it fits our practice’s vision. | R5, GP | |
| I was not really paying attention to that term [Positive Health], but I do notice that what I have been working on and what I really like fits that bill. | R6, GP | |
| Yeah well, that’s why I say it has gotten the name Positive Health. Especially that model Positive Health what has been worked out. (…) I think we were doing that already and it’s of course really beautiful if you can cast that into a model. | R11, GP | |
| *On how Positive Health is not always a match* |  | |
| Look if it’s in your nature to be uptight and to just stick to the rules. Well, you can work with Positive Health all you want, [but] your character gets in your way then. So I do wonder a little, I think it also depends a bit on how you are. | | R2, practice nurse |
| I think if you’re told to function in a certain way and you don’t have affinity for it. Well. Then I think such a Positive Health project won’t work. | R7, X | |
| *On Positive Health and the agreements with the health insurer* |  | |
| The moment you are at the negotiating table with an insurer and you say we’re embracing the concept Positive Health that can have its advantages, right. The moment this aligns with the stakes of the insurer, then you understand each other better. | R10, nurse specialist | |
| You can join it and also use it as a frame for conversations with others in the field, because everyone has learned a little and heard about Positive Health and which added value it can offer. You visualize the same concept and image more quickly. ‘Oh you are adopting Positive Health? All right, good, then I know what it is.’ That’s much shorter said than we think it’s important to give space, ask additional time for the patients, looking at dimensions of life. It’s in fact also a matter of definition and you speak a little bit the same language. You don’t have to explain everything anymore. | R10, nurse specialist | |
| In that time [the health insurer] came by. They bumped into the practice like: ‘They are doing something nice, they’re trying to do something different’. And then the template of Positive Health came in the picture. Like: ‘couldn’t you do it like this? Or could that be something that could be supportive?’ So we were working on it already a little bit, it just didn’t have a name at ours’. | R11, GP | |
| Theme 2: Giving substance to Positive Health in practice | | |
| *On how they imagine the future* | |  |
| I hope that together with all cooperating partners we can set a new standard of how care should be arranged; with bigger practices, really more time for the patient, where one really looks at what’s needed in this situation versus what pill gives a short relief of symptoms? (…) That we can make a case for all those other practices. | | R1, practice manager |
| I hope that this [Positive Health] will be adopted in multiple practices, because you see that it matters so much when time is being spent on the people and if the people aren’t treated as a number, but as patient. | | R3, practice nurse |
| I think specifically for our practice that we can’t do it differently anymore. I consider this such a nice way of working. That doctors-centred and problem-oriented way of working I would not want that anymore. So it’s not even an option for me at all to work in a practice that doesn’t work like this. | | R6, GP |
| At the moment it’s an experiment, a pilot. Eventually it will have to show whether this way of providing care isn’t more expensive than the traditional way of care provision. And actually, we assume that looking at the bigger picture, you could realise a cost reduction. That you can avoid the more expensive care. But that will have to show. And I really hope that this will be the case, because this is a much nicer way of working, that according to me has a lot of added value for the patient, and also for the community in general. (…) If we, as a practice, can keep contributing to that in this way, yes please. | | R10, nurse specialist |
| *On how they express/ promote Positive Health* | |  |
| It’s not as if we continuously have those pillars of Positive Health lying next to us. But it’s more interwoven in the everyday conduct, daily processes, daily communication. | | R1, practice manager |
| We have a healthy lunch together, we make salads, we’re on the right track with food. We’re taking a walk at lunchtime. It is also the image towards the outside. So actually as a team, as a practice you’re engaging with Positive Health towards the outside world in how you deal with everything. Of course we [the practice nurses] are also part of that. (R2, practice nurse) | | R2, practice nurse |
| [On adopting Positive Health] We’re also trying to carry out, so to say, as a practice that we think connection, self-direction are important. Time and calmness and attention. We try to carry that out too. That’s an image to the outside that has been changed. | | R5, GP |
| [On adopting Positive Health] It’s not always concrete. But it’s more interwoven in the way of working so to say. So then, after all, you end up with these concepts of attention, self-direction, resilience, connection, and so forth. | | R5, GP |
| That the team is like: ‘Well the doctor stands for this, we stand for this.’ If you look at the website. Everyone introduces themselves on the website, tells something about their passion for their profession, something personal and hobbies. | | R7, X |
| We’re trying to setting the right example and with pride. Not hiding yourself or walking somewhere where you don’t run into people. No, if you run into patients, just wave. They say ‘are you going on a nice stroll?’ Saying ‘Well, we have to stretch our legs! It’s good for us.’ And people love seeing that. | | R7, X |
| We lunch together. We walk together. We eat salad instead of, well we also eat other things. We try to coach each other in the direction of healthy behaviour. | | R9, practice nurse |
| *On job enjoyment* | |  |
| I: What makes your joy in work go up? R1: Well, I was thinking about it yesterday. Look. I have worked for ten years at [university of applied sciences] as teacher and I was so bound to rules and to check marks. Now, I have the complete freedom to do what I think is right. And of course I then discuss with R11, with R5, with collaboration partners what I’m going to do, but I am just completely free. | | R1, practice manager |
| [On why she found joy in work] This is more like how I want to work. I don’t want to hold people off. And look, you shouldn’t let people wait a whole week before they can see the GP. That made me, I did not like that, I don’t want that. | | R3, practice nurse |
| What’s most important to me, is that I experience and get a carte blanche concerning how carry out and organize my practice. I can give substance to it completely as I want to. Also my scheduling. Even the time I spend on patients is not strained by a watchful eye who checks whether I do enough or see enough patients. It’s completely free. And I love that, because that’s how I ideally work. (R4, nurse specialist) | | R4, nurse specialist |
| The man told me during the conversation, just before the long function [test] started. ‘R4, I have smoked a lot more than normal.’ I could have done two things. I could have said: ‘Well we’ll see that in the function test. We’ll see that it went down.’ (…) I did it differently. At a certain moment I asked. ‘Tell me why you smoked so much more.’ (…) He told me the story about the fact that his wife had been diagnosed with some sort of cancer a week ago, and that it had already spread through here entire body. So I pushed the spirometer aside. For 50 minutes I talked with that man about just that. Well, in that [other] practice I was being told off for doing that. (…) Being open towards someone’s input having an ear and an eye for a signal someone gives, being able to and allowed to delve into that within the work environment you reside, that’s a really important part of my job enjoyment. | | R4, nurse specialist |
| That I can express my vision on lifestyle here (…) That’s really important in my job enjoyment. (…) The moment I cannot tell someone that I don’t think that a cholesterol lowering [pill] is such a good option, but [when] I should give it without discussing other options, I don’t just consider myself a good doctor, because I would definitely advise my family members and myself something else. | | R6, GP |
| What I really like is that I feel that responsibility a little less and I don’t know whether that’s Positive Health or just the fact that I have already been a GP for six years (…) But that you can put that responsibility with the patient. That I can indeed say: Listen these are the options as far as I am concerned and I’ll hear from you what suits you. | | R6, GP |
| I think it’s important that I’m not checked upon the entire time, because that will, that feels like I kind of have to defend myself and what I’m doing the entire time. And I don’t have to do that. I think it’s nice to get that freedom. | | R8, practice nurse |
| [On job enjoyment] The most important part is being myself and also that no one will tell me at some point that I can’t hug anyone anymore. Let me do my thing, then I’m the best practice nurse. | | R9, practice nurse |
| [On job enjoyment] I consider autonomy an important part of my work. Equal conversations I think is important in my job enjoyment. Acceptable working conditions contribute to it as well. Recognition. And of course the satisfaction of being able to help people and getting the space to give that substance. | | R10, nurse specialist |
| For example, I can determine how often I see a patient. (…) The proverbial old lady in the nursing home who just wants to let off steam about everything that her life doesn’t offer anymore. Being able and allowed to offer a sympathetic ear for this. Nice! Feeling the freedom to go on a walk with a patient. To walk outside together and talk about his problems. Amazing! I am not saying that you should always do this in any case, but having the possibility, the choice, the autonomy and the freedom to give substance to that. That’s nice and important. | | R10, nurse specialist |
| When you connect with people, you’re alive right. When you’re carrying out protocols, then you become a vegetable. | | R11, GP |
| [On job enjoyment] That you can carry it out with gusto. That I can visit patients by bike, that you walk in the afternoon. That once in a while you don’t work. So, that you don’t have a fulltime job, but have time off too. And that you do that, just because it’s nice. That that’s allowed. | | R11, GP |
| Theme 3: Changing financial and organizational structures | |  |
| *On the new financial agreements* | |  |
| We’re not financially dependent on what choices we make. That gives a lot of peace. | | R1, practice manager |
| Personally I am a big fan of this way of financing because the pressure to perform treatments completely falls away, right. That could maybe benefit the patient care. But it depends a lot on your vision right, because they [the practice owners] are obviously making a considerable concession on their own income. | | R4, nurse specialist |
| The happy professional is also very important. You notice with respect to the atmosphere within the practice that there’s space to take your time for patients. That means that there’s less pressure on the care provider, which makes the ambience in general more relaxed. I also know practices where the profit targets are more on the foreground and that results in a different ambience. | | R10, nurse specialist |
| *On more having more time* | |  |
| What we do differently? Well, just literally more time. More time for the patients, for each other, for ourselves, for a nice lunch. | | R1, practice manager |
| Because it [more time] literally brings peace. That literally shifts the focus from the schedule to the patient. That just has to do with attention. | | R5, GP |
| You’ll say: ‘Well I see you’re coming for knee complaints, how is your knee? Shall we take a look at that knee?’ And then mostly having the patient outside within ten minutes. Without discussing: ‘How does this affect your situation at home and what does this mean for your work?’ So the time is really important, because only then, at least I feel the space only then to move a patient towards positivity. | | R6, GP |
| You get the opportunity to occupy yourself with the patients. When I’m taking blood, there’s no pressure on me. Like: ‘I have to move on, bummer.’ If you have more time, then you’ll get more of my time. That’s what it is. And another has to wait a bit longer. I think that’s very important. | | R9, practice nurse |
| Positive Health is quite a comprehensive model and you just need the time to be able to address all dimensions. That means that you need to take time to touch upon various dimensions with a patient. The moment that you have a consult of ten minutes, then you are limited in what you can ask and what you can focus your attention on. The moment you have more space for that, well then it’s a lot easier. | | R10, nurse specialist |
| *On job enjoyment* | |  |
| When someone comes with a headache, for example. Maybe there’s something that you worry about. Or with respect to sleeping badly, what’s behind that? What’s the cause of that? Because if you just have the time for the people, often you’ll hear these things. And here, there’s time to go into these things. I really like that. | | R3, practice nurse |
| [On job enjoyment] Well first and foremost, it’s the secondary conditions. So the fact that we have the possibility, and the peace too, to take more time for the patient. That we don’t have to worry about the financial consequences of that. We can fully focus on quality of care. | | R5, GP |
| [On job enjoyment] Number one anyway is that time. It’s just nice to have. I am working much more relaxed and am a lot less tired at night. (…) I have worked in practices, well. The work pressure was so high that at the end of the day I did not know what I had done anymore. | | R6, GP |
| The walking (…) that’s also really chill. Or just sitting here in the courtyard, shortly talking with each other. I notice that it’s less fun if you’ve been this busy that you’ve just been working in your room by yourself. | | R7, X |
| [On how PH affected her job enjoyment] Normally, you have ten minutes for a patient to provide treatment, taking blood, placing an injection. When I know that there’s no one else sitting in the lab, I’ll make it twenty so to say. I can choose that myself. If we wouldn’t have had Positive Health. I mean, if you plan everything for ten minutes, you just don’t have the time to really chat with people or to go for a stroll once in a while. | | R8, practice nurse |
| [On job enjoyment] The doctor, he just always has to have circles under his eyes, right, he’s just always working. But that is a very bad example by us, the ones who are being watched. They should be as happy as possible right. That’s what we should do, demonstrate it well. So that’s an important aspect of the different financing; also being able to pay attention to your own health in fact. That you may have more time for that, more attention. | | R11, GP |
